# 

**Published:** 1901-06

**Authors:** 


### The Library of the Bristol Medico-Chirurgical Society.

*The following donations have been received since the publication of the List in March:*

*May 31st, 1901.*

| | |
|---|---|
| H. A. Benham, M.D. (1) ... ... ... ... ... ... | 10 volumes. |
| J. Michell Clarke, M.D. (2) ... ... ... ... ... ... | 2 ,, |
| W. R. Cossham, M.D. (3) ... ... ... ... ... ... | 13 ,, |
| F. H. Edgeworth, M.B. (4) ... ... ... ... ... ... | 6 ,, |
| The Faculty of Physicians and Surgeons of Glasgow (5) ... ... ... ... ... ... ... ... | 2 ,, |
| L. M. Griffiths (6) ... ... ... ... ... ... ... ... | 2 ,, |
| The Representatives of the late J. E. Prichard, M.B. (7) ... ... ... ... ... ... ... | 185 ,, |
| Bertram M. H. Rogers, M.D. (8)... ... ... ... | 70 ,, |
| James Swain, M.D.... ... ... ... ... ... ... ... | 19 ,, |

Three framed pictures have been presented by Mr. Arthur W. Prichard, and unbound periodicals have been received from the Representatives of the late Dr. J. E. Prichard and from Dr. Shingleton Smith.

### FORTIETH LIST OF BOOKS.

The titles of books mentioned in previous lists are not repeated.

The figures in brackets refer to the figures after the names of the donors, and show by whom the volumes were presented. The books to which no such figures are attached have either been bought from the Library Fund or received through the *Journal*.

| | | | |
|---|---|---|---|
| *Anæsthetics Committee, Report of the*—British Medical Association ... | | ... | 1900 |
| **Andrew, J. G.** ... | *Tubercular Disease of the Hip-joint*... ... ... ... ... ... | | 1901 |
| **Archambaud, P.** | *Traitement de la Coxalgie* ... ... ... ... ... ... ... ... | | 1901 |
| **Armstrong, J.** ... | *On Puerperal Fever* ... ... ... ... ... ... | (3) 2nd Ed. | 1819 |
| **Arnott, J.** ... ... | *Contributions to Practical Medicine and Surgery* ... | (7) | 1864 |
| **Atkinson, J.** ... | *Medical Bibliography (A. and B.)* ... ... ... ... | (3) | 1834 |
| **Auld, A. G.** ... | *Selected Researches in Pathology* ... ... ... ... ... ... | | 1901 |
| **Ayre, J.** ... ... | *On Disorders of the Liver* .. ... ... | (3) 2nd Ed. | 1821 |
| **Baillie, M.**... ... | *Morbid Anatomy* ... ... ... ... ... ... | (3) 4th Ed. | 1812 |
| **Barr, T.** ... ... | *Diseases of the Ear* ... ... ... ... ... ... ... | 3rd Ed. | 1901 |
| **Barton, J. K.** ... | *Records from General Practice* ... .. ... ... ... ... ... | | 1901 |
| **Basham, W. R.**... | *Aids to Diagnosis of Diseases of the Kidneys* ... ... | (8) | 1872 |
| **Belfield, H. M. Lyman, C. Fenger, H. W. Jones, and W. T.** | *The Practical Home Physician and Encyclopædia of Medicine* ... | | [N.D.] |
| **Bell, Sir C.** ... | *The Nervous System of the Human Body* | (7) 3rd Ed. | 1844 |
| **Bellini, M.** ... | *La Résistance du Crâne* ... ... ... ... ... ... ... ... | | 1900 |
| **Bernheim, [H.]** ... | *De la Suggestion* ... ... .. ... ... ... ... ... | (4) | 1888 |
| **Berry, J.** ... ... | *Diseases of the Thyroid Gland* .. ... ... ... .. ... | | 1901 |
| **Billroth, T.** ... | *Surgical Pathology and Therapeutics* (Tr. by C. E. Hackley) ... ... ... ... ... ... ... ... ... | (7) | 1875 |
| **Binet et C. Féré, A.** | *Le Magnétisme animal* ... ... ... ... | (4) 2e Éd. | 1838 |

**Bowman, R. B. Todd and W.** *The Physiological Anatomy and Physiology of Man.* Parts II., III. ... ... ... ... ... ... (7) 1845

**Bucknill, J. C.** ... *Care of the Insane* .. ... ... ... ... ... ... ... ... (8) 1880

**Burns, A.** ... ... *Diseases of the Heart* ... ... ... ... ... ... ... (7) 1809

*Catalogue of the Library of the Faculty of Physicians and Surgeons of Glasgow.* 2 vols. ... ... ... ... ... ... (5) 1885–1901

*Catalogue of the Library of the Royal Medical and Chirurgical Society of London* ... ... ... ... ... ... ... ... ... ... (8) 1844

**Chambers, T. K.** *Lectures: Chiefly Clinical* ... ... .. (8) [3rd Ed.] 1864

**Chowry-Muthu, D. J.** *Some Results of Sanatorium Treatment of Phthisis*... 1901

**Copeland, T.** ... *On the Rectum and Anus* ... ... ... ... (3) 3rd Ed. 1824

**Corvisart, J. N.**... *Diseases of the Heart* (Tr. by C. H. Hebb) ... .. (7) 1813

**Crowther, B.** ... *On White Swelling, &c.* ... ... ... ... New Ed. 1808

**Curling, T. B.** ... *Diseases of the Rectum* ... ... ... ... (8) 3rd Ed. 1863

**Currie, J.** ... ... *Medical Reports on the Effects of Water in Fever and other Diseases.* 2 vols. ... ... ... (7) 3rd Ed. 1804

**Donkin, A. S.** ... *Diabetes and Bright's Disease* ... ... ... ... ... (8) 1871

**Duncan, A. (Jun.)** *The Edinburgh New Dispensary* ... ... (7) 2nd Ed. 1804

**Fenger, H. W. Jones, and W. T. Belfield, H. M. Lyman, C.** *The Practical Home Physician and Encyclopædia of Medicine* (7) [N.D.]

**Féré, A. Binet et C.** *Le Magnétisme animal* ... ... ... (4) 2e Éd. 1838

**Fischer, L.** ... *Infant-feeding in its Relation to Health and Disease* ... 1901

**Forel, A.** ... ... *Der Hypnotismus* ... ... ... ... ... ... ... ... (4) 1889

**Fox, R. H.**... ... *William Hunter, Anatomist, Physician, Obstetrician* (1718-1783) 1901

**Freyer, P. J,** ... *Stricture of the Urethra* ... ... ... ... ... ... ... 1901

**Garrod, A. B.** ... *Materia Medica and Therapeutics* (Ed. by E. B. Baxter) ... ... ... ... ... ... (7) 7th Ed. 1879

**Geddes and J. A. Thomson, P.** *The Evolution of Sex* ... ... [3rd Ed.] 1898

**Gee, S.** ... ... *Auscultation and Percussion* ... ... ... ... ... (8) 1870

**Golebiewski, E.**.. *Diseases Caused by Accidents* (Tr. and Ed. by P. Bailey)... ... ... ... ... ... ... ... ... ... 1900

**Grasset, J.**......... *Leçons sur deux Cas d'Hystérie* ... ... ... ... (2) 1890

**Guthrie, G. J.**.... *On Diseases and Injuries of Arteries* ... ... ... (3) 1830

**Haden, C. T.** ... *On the Colchicum Autumnale* ... ... ... ... ... ... 1820

**Hammond, W. A.** *Military Medical and Surgical Essays* ... ... ... (8) 1864

**Hewer, Mrs. L.**... *Our Baby* ... ... ... ... ... ... ... ... 7th Ed. 1901

**Hewitt, G.**... .. *Severe Vomiting during Pregnancy* ... ... ... ... (8) 1890

**Higginbottom, J.** *Additional Observations on the Nitrate of Silver* ... (7) 1850

**Hime, T. W.** ... *Practical Guide to the Public Health Acts* ... 2nd Ed. 1901

**Howe, S. G. [et al.]** *The Causes of Idiocy* ... ... ... ... ... ... (8) 1858

**Hunter, W.** ... *Pernicious Anæmia* ... ... ... ... ... ... ... ... 1901

**Jones, H. M.** ... *Practical Points in Gynæcology*... ... ... ... ... ... 1901

**Jones, and W. T. Belfield, H. M. Lyman, C. Fenger, H. W.** *The Practical Home Physician and Encyclopædia of Medicine* ... (7) [N.D.]

**Kemp, W.** ... ... *A Brief Treatise of the Pestilence* ... ... ... ... (6) 1665

**Kennedy, R. H.**... *Epidemic Cholera* ... ... ... ... ... (7) 2nd Ed. 1846

**King, P.** ... ... *Bath Waters* ... ... ... ... ... ... ... ... ... ... [N.D.]

**Kourkine, E. Ossipow, I. Popow et P.** *La Médecine du Zemstwo en Russie*... ... ... ... ... ... ... ... ... ... (1) 1900

**Lambe, W.** ... *On Scirrhous Tumours and Cancerous Ulcers* ... ... (7) 1809

**Law, W.** ... ... *On Derangement of the Digestive Organs* (3) 2nd Ed. 1829

**Leftwich, R. W.** *An Index of Symptoms* ... ... ... ... ... ... 2nd Ed. 1901
**Lehmann, A.** ... *Die Hypnose* ... ... ... ... ... ... ... ... ... ... (4) 1890
**Lockwood, C. B.** *Appendicitis* ... ... ... ... ... ... ... ... ... ... 1901
**Loeb, J.** ... ... *Comparative Physiology of the Brain and Comparative Psychology* ... ... ... ... ... ... ... ... ... ... 1901
**Lugol, [J. G. A.]** *On Iodine in Scrofulous Diseases* (Tr. by W. B. O'Shaughnessy) ... ... ... ... ... ... ... (7) 1831
**Lyman, C. Fenger, H.W. Jones and W.T. Belfield, H.M.** *The Practical Home Physician and Encyclopædia of Medicine* ... (7) [N.D.]
**Maguire, R.** ... *The Harveian Lectures on Pulmonary Tuberculosis* ... 1901
**Martin, J.** ... ... *De l'Atrophie du Nerf optique* ... ... ... ... (2) 1890
**Martindale, and W. W. Westcott, W.** *The Extra Pharmacopœia* 10th Ed. 1901
**Martinet, [A. J. B.] Parent-Duchatelet et L.** *Recherches sur l'Inflammation de l'Arachnoïde* ... ... ... ... ... ... ... ... (8) 1821
**Monti, A.** ... ... *Modern Pathology* (Tr. by J. J. Eyre) ... ... .. ... 1900
**Moullin, C. M.** ... *When to Operate in Inflammation of the Appendix* 2nd Ed. 1901
**Orton, R.** ... ... *On the Epidemic Cholera of India* ... ... (3) 2nd Ed. 1831
**Ossipow, I. Popow et P. Kourkine, E.** *La Médecine du Zemstwo en Russie* (1) 1900
*Pamphlets.* 3 vols. ... ... ... ... .. ... ... ... ... ... ... (7) [V.D.]
**Parent-Duchatelet et L. Martinet, [A. J. B.]** *Recherches sur l'Inflammation de l'Arachnoïde* ... ... ... ... ... ... ... (8) 1821
*Parish Leech, The.* *By a "Parish Doctor"* ... ... ... ... ... ... (6) 1870
**Parkes, E. A.** ... *On Urine* .. ... ... ... ... ... ... ... ... ... (8) 1860
*Pharmacopœia in usum Nosocomii Londinensis Sancti Georgii* ... ... (4) 1768
**Philip, A. P. W.** *On Febrile Diseases.* Vol. II. ... ... (7) 3rd Ed. 1813
**Pilcher, G.** ... *Diseases of the Ear* ... ... ... ... ... ... (7) 2nd Ed. 1842
**Pitta, N. C** ... *On the Influence of Climate on the Human Species* ... (7) 1812
**Popow et P. Kourkine, E. Ossipow, I.** *La Médecine du Zemstwo en Russie* (1) 1900
**Porter, G. H.** ... *Surgical Reports* ... ... ... ... ... .. ... (7) 1869
*Questions (3,500) on Medical Subjects, arranged for Self-Examination* 3rd Ed. 1901
**Rabagliati, A.** ... *Aphorisms, Definitions, Reflections, and Paradoxes* ... 1901
**Richet, C.** ... ... *Experimentelle Studien auf dem Gebiete der Gedankenübertragung.* (Deutsche Ausgabe von A. F. von Schrenck-Notzing) ... ... ... ... ... ... (4) 1891
**Ridge, B.** ... ... *Health and Disease* ... ... ... ... ... ... (8) 2nd Ed. 1859
**Roberts, A.** ... *The Use and Abuse of the Harrogate Mineral Waters* 3rd Ed. 1901
**Santi, P. R. W.** *Golden Rules of Aural and Nasal Practice* ... ... ... [1901]
**Scherbakov, A.** ... *Les Stations de Boues minérales de la Russie d'Europe* (1) 1897
**Senn, N.** ... ... *Principles of Surgery* ... ... ... ... ... 3rd Ed. 1901
**Sewill, H.** ... ... *Dental Surgery* (Ed. by W. J. England and J. S. Sewill) 4th Ed. 1901
**Scudamore, C.** ... *On Gout, Gravel, and Disorders of the Digestive Organs* (3) 4th Ed. 1823
**Scudder, C. L.** ... *The Treatment of Fractures* (assisted by F. J. Cotton) 2nd Ed. 1901
**Sims, J.** ... ... *Pathology of the Brain* .. ... ... ... ... ... ... (7) 1835
**Spillan, D.** ... ... *A Supplement to the London, Edinburgh and Dublin Pharmacopœias* ... ... ... ... ... ... ... ... (3) 1830
**Thomson, P. Geddes and J. A.** *The Evolution of Sex* ... [3rd Ed.] 1898
**Todd and W Bowman, R. B.** *The Physiological Anatomy and Physiology of Man.* Parts II., III. ... ... ... ... ... ... (7) 1845

**Tubby, A. H.** ... *Clinical Lectures on the Various Forms of Intra-Abdominal Suppuration* ... ... ... ... ... ... 1901
**Turner, T.** ... ... *A Practical Treatise on the Arterial System* ... ... ... 1825
**Wardell, J. R.** ... *Contributions to Pathology and the Practice of Medicine* (8) 1885
**Watteville, A. de.** *Medical Electricity* ... ... ... ... ... ... ... ... (8) 1878
**Westcott, W. Martindale and W. W.** *The Extra Pharmacopœia* 10th Ed. 1901
**Wilde, W. R.** ... *Aural Surgery* ... ... ... ... ... ... ... ... ... (7) 1853
**Williams, P. W.** *Diseases of the Upper Respiratory Tract* ... 4th Ed. 1901
**Wood, W.** ... ... *Cerebral Science* ... ... ... .. ... ... ... .. ... 1901
**Yearsley, J.** ... *Deafness Practically Illustrated*... ... ... (7) 4th Ed. 1854
**Zavitziano, S. C.** *Service des Enfants trouvés de Notre-Dame de Péra* ...[1901]

### TRANSACTIONS, REPORTS, JOURNALS, &c.

American Dermatological Association, Transactions of the ... ... ... 1901
American Gynæcological and Obstetrical Journal, The ... Vol. XVII. 1900
American Journal of Obstetrics, The ... ... ... ... ... Vol. XLII. 1900
American Pediatric Society, Transactions of the ... ... Vol. XII. 1900
American Public Health Association, Reports and Papers of the Vol. XXVI. 1901
Archives de Neurologie ... ... ... ... ... ...2e Sér., Tomes IX., X. 1900
Archives of Pediatrics ... ... ... ... ... ... ... ... ... Vol. XVII. 1900
Archives provinciales de Chirurgie ... ... ... ... ... Tome IX. 1900
Bristol Medico-Chirurgical Journal, The ... ... ... ... Vol. XVIII. 1900
Bristol Port Sanitary District.—Annual Report of the Medical Officers of Health for 1900... ... ... ... ... ... ... ... ... ... 1901
British Journal of Dermatology ... ... ... ... ... ... Vol. XII. 1900
Canadian Practitioner and Review, The ... ... ... ... Vol. XXV. 1900
Centralblatt für innere Medicin ... ... ... ... ... ... ... ... ... 1900
Cincinnati Lancet-Clinic, The ... ... ... ... ... Vol. LXXXIV. 1900
Clinical Journal, The ... ... ... ... ... ... ... ... ... Vol. XVII. 1901
College of Physicians of Philadelphia, Transactions of the 3rd Ser., Vol XXII. 1900
Congrès (XIIIe) international de Médecine, Paris, 1900.—Comptes Rendus ... ... ... .. ... [Vols. I., V., VII., XI., XIII. 1901]
Congrès (XIIe) international de Médecine, Moscou, 1897.—Comptes Rendus. 7 vols. in 8 ... ... ... ... ... ... ... (1) 1898-1900
Grèce médicale, La ... ... ... ... ... ... ... ... ... ... ... ... 1900
Hospitals—
Johns Hopkins Hospital Reports, The ... ... ... Vol. VII. 1899
Indian Medical Gazette, The ... ... ... ... ... ... ... ... Vol. XXXV. 1900
International Medical Magazine ... ... ... ... ... ... ... Vol. IX. 1900
Iowa State Medical Society, Transactions of the ... ... Vol. XVIII. 1900
Johns Hopkins Hospital Bulletin, The ... ... ... ... .. ... Vol. XI. 1900
Journal d'Hygiène ... ... ... ... ... ... ... ... ... ... Vol. XXV. 1900
Journal of Cutaneous and Genito-Urinary Diseases ... Vol. XVIII. 1900
Journal of Laryngology, Rhinology, and Otology ... ... Vol. XV. 1900
Journal of Mental Science, The ... ... ... ... ... ... Vol. XLVI. 1900
Journal of Nervous and Mental Disease, The ... ... ... Vol. XXVII. 1900
Journal of the American Medical Association, The ... ... Vol. XXXV. 1900
Journal of Tropical Medicine, The ... ... ... ... ... Vol. III. 1900
Laryngoscope, The ... ... ... ... ... ... ... ... ... ... Vol. VIII. 1900
Librarians, Transactions and Proceedings of the Conference of, 1877 ... 1878

Llangyfelach Rural District Council—Annual Report on the Llandilo-Talybont Division for 1900 ... ... ... ... . . ... ... ... 1901
Medical Age, The ... ... ... ... ... ... ... ... ... Vol. XVIII. 1900
Medical Annual, The ... ... ... ... ... ... ... ... ... ... 1901
Medical Examiner, The ... ... ... ... ... ... ... ... ... Vol. I. 1900
Medical News, The ... ... ... ... ... ... ... ... Vol. LXXVII. 1900
Medical Record, The ... ... ... ... .. ... ... ... Vol. LVIII. 1900
Medical Review ... ... .. ... ... ... ... ... ... ... Vol. III. 1900
Medical Transactions published by the College of Physicians in London (3) Vol. VI. 1820
Merck's Annual Report [on the New Medicinal Preparations] for 1900 1901
Monthly Cyclopædia of Practical Medicine, The ... N.S., Vol. III. 1900
Montreal Medical Journal, The ... ... ... ... ... ... Vol. XXIX. [1900]
Newquay, The 12th Annual Report on the Health and Sanitary Condition of the Town and Urban District of, for 1900
Ophthalmological Society of the United Kingdom, Transactions of the Vol. XX. 1900
Progrès médical, Le ... ... ... ... ... ... 3e Sér., Tomes XI., XII. 1900
St. Louis Medical and Surgical Journal, The... Vols. LXXVIII., LXXIX. 1900
St. Petersburger medicinische Wochenschrift ... ... . ... ... ... 1900
Sanitary Institute, Transactions of the.—General Index to Vols. I.-XXI., 1876-1900 [1901]
Smithsonian Institution, Annual Report of the, for 1898... ... ... ... 1899
Therapeutic Gazette, The ... ... ... ... ... ... ... ... Vol. XXIV. 1900
Travaux pratiques d'Obstétrique et de Gynécologie ... ... ... [1899, 1900]
Treatment ... ... ... ... ... ... ... ... ... .. ... ... Vol. IV. 1901
University Medical Magazine ... ... ... ...Vols. XII., XIII. 1899-1901
West London Medical Journal ... ... ... ... ... ... ... Vol. V. 1900
Zeitschrift für Krankenpflege ... ... ... ... ... ... ... ... ... ... [1900]

### Local Medical Notes.

### GLOUCESTER, SOMERSET, AND WILTS BRANCH OF THE NATIONAL ASSOCIATION FOR CONSUMPTION.

The following is an outline of the plans of the proposed Sanatorium at Winsley, subject to the approval and possible slight modification by a sub-committee, consisting of Dr. L. A. Weatherly (Chairman of Executive), Dr. Watson Williams (Hon. Treasurer), Dr. Michell Clarke (Hon. Sec.), Dr. Davies (Medical Officer of Health for Bristol), Dr. Tubb Thomas (Medical Officer of Health for Wiltshire), Dr. Symons (Medical Officer of Health for Bath), Dr. Pruen (of the Cotswold Sanatorium), and Dr. Thurnam (of Nordrach-on-Mendip Sanatorium):

In designing the building the plans of the principal Continental Sanatoria have been consulted, and the leading features in them which experience has proved to be desirable have been adopted.

The general arrangement consists of a long main building with wings at both ends, set on the south side at an obtuse angle, thus securing for the various apartments the maximum amount of sunshine with the least exposure to the easterly winds.

On the north side the wings project at right angles to the main building, and in addition there is a large projection in the centre containing the principal entrance and hall; the kitchen and offices, together with the dining-hall over, are contained in a building separated entirely from the Sanatorium proper, and approached by a covered way and bridge.

The building has three floors, each ten feet high, consisting of a wide corridor extending nearly the entire length of the north side, with the rooms on the south; at the junction with the wings the corridor is continued, but in the centre with rooms on either side.

The ground floor contains a spacious entrance-hall, with bureau, staircase, reception-room, library, large cloak-rooms, wardrobes, and disinfecting-room; one wing is devoted to the use of the Medical Officer, and contains his dining-room, study, consultation-room, and laboratory and dispensary. Suitable apartments are provided in the other wing for the Matron, and immediately adjoining them are the linen and store rooms.

In addition to the principal staircase, others are provided at each end of the north corridor, affording easy access to all parts of the building.

A passenger-lift, capable of containing a bed or reclining chair, is also provided.

The two projecting buildings at the end of the north front contain the lavatories, bath-rooms, and w.c.'s; ample provision being made for both sexes. Especial care has been taken to entirely disconnect these from the main building; this has been done by means of a ventilated passage.

The first and second floors are given up to the bedrooms, and additional rooms have been provided in the roof for the use of the female staff.

The building will be capable of accommodating 60 patients.

A wide verandah or liegehalle runs along the south side and returns round the two wings; and additional verandahs are provided on the north side, sheltered from the east winds by the projecting buildings.

The building is to be erected with rough stone obtained from the site, and the walls will be impervious to moisture by a facing of a cement stucco.

The woodwork will be painted green and white, and the roofs will be covered with red tiles, and it is hoped that in this way a pleasing effect will be obtained.

### BRISTOL.

University College.—The following appointments have been gained by former students of the Bristol Medical School:—Mr. J. E. Long, M.R.C.S., L.R.C.P., House Surgeon to the Torbay Hospital and Provident Dispensary, Torquay; Mr. L. A. Moore, M.R.C.S., L.R.C.P., House Surgeon to the Warneford, Leamington, and South Warwickshire Hospital; Mr. S. V. Stock, M.B., B.S. Lond., has passed the primary examination for the Fellowship of the Royal College of Surgeons of England.

On Monday, May 13th, a Medical Students' Dance was held in the New Hall of the College. About 220 persons attended, and the dance proved a great success.

Bristol General Hospital.—Mr. J. W. Malim, B.A., M.B., B.C. Cantab., has been appointed Casualty Officer, and Mr. A. F. Martin, M.B., Ch.B. Vict., Assistant House Surgeon.

Bristol Royal Hospital for Sick Women and Children.—We are glad to hear that the Bazaar held to obtain funds for the above institution, and opened by Earl Roberts on May 1st, was very successful, and that upwards of £3,000 was gained for the hospital funds after deducting expenses.

Appointments.—William Cotton, Esq., M.A., M.D., D.P.H., was appointed Medical Officer H.M. Prison, Bristol, in March. Frederick Hudson Evans, M.R.C.S. Eng., L.R.C.P. Lond., was appointed Medical Superintendent at the Mendip Hills Sanatorium for Consumption, Hill Grove, Wells, Somerset, *vice* Charles Joseph Whitby, B.A., M.D. Cantab., resigned.

The Lord Mayor's Hospital Sunday Fund.—The list of contributions to this fund collected in the various churches and chapels of the district for the year 1901, the third year in which a Hospital Sunday has been observed, are now to hand. The following tabular statement of the amounts received and their source is of interest:—

| | £ | s. | d. | £ | s. | d. |
|---|---|---|---|---|---|---|
| 68 Churches in the city contributed ... ... ... | 924 | 14 | 8 | | | |
| 35 ,, in the country ,, ... ... ... | 163 | 11 | 8 | | | |
| | | | | 1,088 | 6 | 4 |
| 26 Congregational Churches ,, ... ... ... ... ... ... | | | | 221 | 15 | 4 |
| 18 Baptist Chapels ,, ... ... ... ... ... ... | | | | 116 | 0 | 9 |
| 30 Wesleyan ,, ,, ... ... ... ... ... ... | | | | 90 | 6 | 9 |
| 23 United Methodist Free Churches contributed ... ... ... | | | | 40 | 3 | 9 |
| 6 Roman Catholic Churches ,, ... ... ... | | | | 21 | 12 | 6 |
| Various other Churches, &c. ,, ... ... ... | | | | 190 | 12 | 4 |
| | | | | £1,768 | 17 | 9 |

The collections were made as usual on the third Sunday in January. It will be remembered that this was the first Sunday after the death of the Queen, and consequently most of the places of worship were unusually crowded, and a correspondingly large collection—for Bristol—was made. The collection in 1900 suffered from the numerous appeals that were made for the sufferers in the war, so that in comparison the amount received this year appears specially good.

At the last meeting of the Committee it was announced that £1,768 17s. 9d. had been received, and to this must be added another guinea. The distribution was made on the plan laid down at the first meeting, and the charities received—

| | £ | s. | d. |
|---|---|---|---|
| Royal Infirmary ... ... ... ... ... ... ... | 704 | 14 | 0 |
| General Hospital ... ... ... ... ... ... ... | 591 | 6 | 0 |
| Children's Hospital... ... ... ... ... ... ... | 218 | 14 | 0 |
| Eye Hospital ... ... ... ... ... ... ... ... | 105 | 6 | 0 |
| Eye Dispensary ... ... ... ... ... ... ... | 5 | 0 | 0 |
| Lock Hospital ... ... ... ... ... ... ... ... | 20 | 0 | 0 |
| Cripples' Home ... ... ... ... ... ... ... | 30 | 0 | 0 |
| | £1,675 | 0 | 0 |

In addition to these, the Bristol Dispensary was sent £35 2s. 2d. and the Medical Mission £1 1s., as the collectors specially requested that the sum sent should be allocated to these charities, so that about £60 was left for expenses and balance.

This is the largest amount yet collected, but it still falls very far short of what a rich city like Bristol should give, though it is possible that the secretaries of the institutions show such activity at other times that the stream is tapped before the collection is drawn.

We are glad to be able to say that the efforts of the Committee to obtain all the collections made on the appointed day were more successful than in previous years, the secretaries of the institutions forwarding to the Fund Committee all amounts sent direct to them. There are still a few more collections to be sent in, and a few country churches and chapels have postponed their collection till the Harvest Festival.

Prevalence of Diphtheria.—The Medical Officer of Health in his report to the Health Committee calls attention to the prevalence of diphtheria in the parts of the city known as Knowle and Bedminster. In the latter district during the past year there have been 210 cases, as against thirty-four in the previous year; while in Knowle there have been forty-five cases, against six in 1899. The fact that nine cases in as many houses occurred in Redcliff—six of the patients going to one school—led to an examination of ninety-two children who made use of one particular room, and fifteen unsuspected cases were discovered by bacteriological examination. In twelve cases the nose had been examined for the bacilli, which were found present in ten; and in six cases the throat had been examined, and the bacilli found in five of them. None of these cases had previously shown distinctive symptoms, illustrating the great value of the bacteriological method of investigation. Dr. Davies called attention to the great importance of recognising these cases. Nasal diphtheria was specially insidious and liable to be overlooked.

Extension of the Fever Hospital.—When the Fever Hospital at Ham Green was built sufficient room was left for considerable enlargement. There appears to be need for further accommodation, and the following facts were communicated to the Health Committee at its meeting on April 2nd:—Built on the same lines as before, four new wards, to hold fifty-three patients and provide the necessary rooms for nurses, &c., would cost £13,300, or about £232 a bed; but, in addition to this, a further extension of the administration block would be required to afford bedrooms for the nurses and maids. According to the engineer's plans, this could be built at the back of the present block, thereby supplying room for thirteen servants and thirteen nurses—a gain of twenty-five beds, as one room would have to be sacrificed. This would cost £3,100. Designs were also brought before the Committee for a new and larger observation block, containing four wards for one bed and two wards for three beds, each at a cost of £3,260. In addition to this, £1,400 would have to be spent on water supply, drainage, electric light, and other fittings. A proposed water tower would cost £1,750, and the cost of loan and supervision is put at £550. Without including the water tower, the cost works out at £342 a bed.

The Eye Dispensary.—During the past year there were 1,776 cases treated in the Eye Dispensary—a very large number when it is remembered that the institution is open only twice a week. The report calls attention to the economical manner in which the dispensary is worked, no porter or dispenser being employed, the staff, with the matron, keeping the books and dispensing what little is required. It is noted that the School Board has lately taken to sending a large number of cases, and the framers of the report express the opinion that that body should provide a medical man with a salary to undertake this important duty.

